# Cell Type Diversity Statistic: An Entropy-Based Metric to Compare Overall Cell Type Composition Across Samples

**DOI:** 10.3389/fgene.2022.855076

**Published:** 2022-04-08

**Authors:** Tanya T Karagiannis, Stefano Monti, Paola Sebastiani

**Affiliations:** ^1^ Institute for Clinical Research and Health Policy Studies, Tufts Medical Center, Boston, MA, United States; ^2^ Bioinformatics Program, Boston University, Boston, MA, United States; ^3^ Division of Computational Biomedicine, Boston University School of Medicine, Boston, MA, United States; ^4^ Department of Biostatistics, Boston University School of Public Health, Boston, MA, United States

**Keywords:** single cell transcriptomic analysis, cell type composition, sample level analysis, sample-to-sample comparison, diversity statistics

## Abstract

Changes of cell type composition across samples can carry biological significance and provide insight into disease and other conditions. Single cell transcriptomics has made it possible to study cell type composition at a fine resolution. Most single cell studies investigate compositional changes between samples for each cell type independently, not accounting for the fixed number of cells per sample in sequencing data. Here, we provide a metric of the distribution of cell type proportions in a sample that can be used to compare the overall distribution of cell types across multiple samples and biological conditions. This is the first method to measure overall cell type composition at the single cell level. We use the method to assess compositional changes in peripheral blood mononuclear cells (PBMCs) related to aging and extreme old age using multiple single cell datasets from individuals of four age groups across the human lifespan.

## Introduction

Tissues are composed of heterogenous cell types that demonstrate differences in biological function (Raj and van Oudenaarden, 2008; Choi and Kim, 2019). Gene expression profiling methods such as single cell RNA-sequencing (scRNA-seq) have made it possible to profile the genome-wide gene expression levels for each single cell of a sample, to account for cell-to-cell variability (Chen et al., 2019; Tanay and Regev, 2017; Choi and Kim, 2019), and to identify and characterize cell types in a given tissue (Jaitin et al., 2014; Macosko et al., 2015; Zheng et al., 2017). ScRNA-seq has been extensively applied in multiple research areas to study cell types and states, as well as cell types compositional changes, across diseases and conditions (Shalek et al., 2014; Baron et al., 2016; Muraro et al., 2016; Villani et al., 2017; Butler et al., 2018; Schaum et al., 2018; Mathys et al., 2019; Velmeshev et al., 2019).

Most methods to analyze cell type composition at a single cell level model each cell type independently from other cell types (Haber et al., 2017; Luecken and Theis, 2019; Hashimoto et al., 2019; Wilk et al., 2020; Zheng et al., 2020; Zhu et al., 2020). For example, changes of peripheral blood mononuclear cells (PBMCs) composition observed between supercentenarians and younger age controls in Hashimoto et al., 2019 were assessed for each cell type *independently* using a Wilcoxon rank sum test. Other studies have taken a similar approach when assessing compositional changes between groups of samples at the single cell level (Haber et al., 2017; Luecken and Theis, 2019; Hashimoto et al., 2019; Wilk et al., 2020; Zheng et al., 2020; Zhu et al., 2020). However, high throughput sequencing data are in fact compositional (Gloor et al., 2016, 2017; Lin and Peddada, 2020). The approach we propose rests on the observation that a sample in scRNA-seq data is composed of cell abundances across cell types that are in constrained proportions, given the total number of cells in the sample (Gloor et al., 2016; Gloor et al., 2017; Lin and Peddada, 2020). In other words, the proportion of cell types within a sample are in fact dependent on each other: if the proportion of one type increases, then others need to decrease (Luecken and Theis, 2019). It is thus necessary to account for this dependency when assessing overall cell type compositional changes across samples. In addition, there is no method that provides a numerical summary of a sample overall cell type composition that can be used to compare samples in different conditions (Luecken and Theis, 2019).

Here, we introduce a statistic to summarize the distribution of the proportions of cell types in a sample. Using three single cell transcriptomic datasets of PBMCs comprising four age groups, we show the utility of this statistic to describe changes in PBMCs composition in aging and extreme old age.

## Materials and Methods

**Cell type diversity statistic.** The statistic makes three assumptions: 1) To make different samples of cells comparable, cell abundances must be normalized based on the total number of cells in a sample; 2) After conditioning on the total number of cells in a sample (Gloor et al., 2017), the cell type composition data is a simplex (Aitchison, 1982), and when the proportion of one cell type changes, the proportion of the other cell types must change as well to maintain the total fixed; and 3) To make the statistic comparable across different cell type resolutions, the statistic must be normalized. Formally, we denote by $p_{i_{s}}=\frac{n_{is}}{n_{s}}$ the proportion of cell type i, for  i=1,…,k in a sample s with $n_{s}$ cells, so that $\overset{k}{\underset{i=1}{\sum }}p_{i_{s}}=1.$

The statistic is adapted from alpha diversity measures applied in ecology and microbiome studies (Whittaker, 1972; Olde Loohuis et al., 2018; Calle, 2019). We measure the overall cell type composition of a sample by the adjusted entropy

$$E_{s}=\frac{-\sum_{i=1}^{k}p_{i_{s}}\log\left(p_{i_{s}}\right)-\log\left(k\right)}{\log\left(k\right)}=\;\frac{-\sum_{i=1}^{k}p_{i_{s}}\log\left(p_{i_{s}}\right)}{\log\left(k\right)}-1$$

In the formula, log(k) is the maximum value of $-\overset{k}{\underset{i=1}{\sum }}p_{i_{s}}\log\left(p_{i_{s}}\right)$ that is reached when $p_{i}=\frac{1}{k}$ for all indexes i,  so that the distribution is uniform. The minimum value of $-\overset{k}{\underset{i=1}{\sum }}p_{i_{s}}\log\left(p_{i_{s}}\right)$ is 0, which corresponds to a mass-point distribution with $p_{i_{s}}=0$ for all indexes i  but one. The adjusted entropy $E_{s}$ therefore ranges between [−1, 0] . A sample with more uniformity in cell type proportions, and hence more variability, will result in a greater cell type diversity statistic and $E_{s}=0$ in a sample with equal proportions of all cell types. A sample with cell type proportions that are skewed towards specific cell types, and less variability, will have a lower statistic and $E_{s}=-1$ when all cells are of one type.

**Data.** To demonstrate the utility of the cell type diversity statistic, we analyzed three single cell transcriptomic datasets of PBMCs representing regular aging and extreme old age. One dataset comprised samples of 7 centenarians from the New England Centenarian Study (NECS) (Sebastiani and Perls, 2012) and 2 younger age controls. We downloaded a publicly available scRNA-seq dataset of PBMCs from 45 younger age controls (van der Wijst et al., 2018), which we will refer to as NATGEN, and a publicly available scRNA-seq dataset of PBMCs from 5 younger age controls and 7 supercentenarians, which we will refer to as PNAS (Hashimoto et al., 2019). We integrated these datasets and stratified the samples into four age groups of the human lifespan: 12 subjects of younger age (20–39), 26 subjects of middle age (40–59), 14 subjects of older age (60–89), and 14 subjects of extreme longevity (100–119). Data processing steps and identification of the 12 cell types are described in the Supplement.

**Application of cell type diversity statistic.** We integrated the datasets to generate a matrix of cell type abundances across samples from all three datasets. We calculated the cell type proportions for each sample such that the sum of the cell type proportions for a particular sample equals to 1. We applied the cell type diversity statistic to different cell type resolutions: 1) based on the proportions of lymphocytes and myeloid cells; and 2) based on the proportions of the 12 lymphocyte and myeloid subpopulations that were detected in the data. For both resolutions, we measured the cell type diversity statistic per sample and compared the differences of the statistics between the four age groups using ANOVA and pairwise T-tests with significance level 0.05.

## Results and Discussion

**FIGURE 1:**
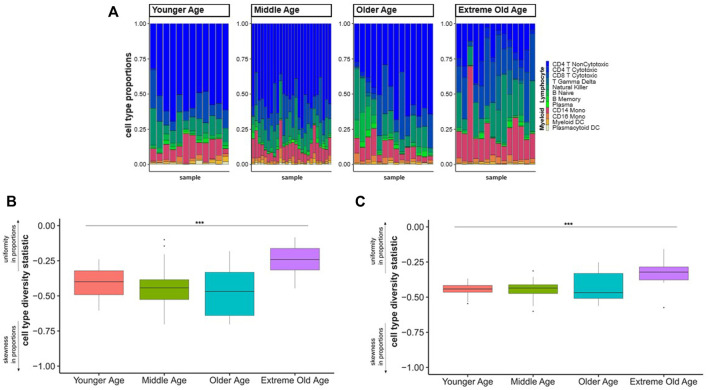
Cell type diversity statistic to summarize PBMCs composition across age groups. **(A)**. Proportions of 12 cell types discovered in scRNA-seq of PBMCs from different age groups. Each bar represents the proportions of lymphocyte (blue-green gradient) and myeloid (red-yellow gradient) cell types (*y*-axis) in a sample. **(B)**. Each boxplot represents the distribution of the diversity statistic of the proportions of lymphocyte and myeloid cells in younger, middle, older, and extreme old age individuals (*x*-axis). The differences of the statistics across age groups were statistically significant (F-test *p*-value = 0.0001873) **(C)**. Each boxplot represents the distribution of the diversity statistic of the proportions of the 12 cell types grouped by younger, middle, older, and extreme old age (*x*-axis). The differences of the statistics across age groups were statistically significant (F-test *p*-value = 0.0001875). The diversity statistic was significantly higher, in the extreme old age group compared to each younger age control group: younger age group (t-test *p*-value = 0.00115), middle age group (t-test *p*-value = 0.00016), and older age group (t-test *p*-value = 0.00363).

We applied the cell type diversity statistic to the cell type proportions from the three scRNA-seq datasets of younger age individuals and centenarians to assess overall compositional changes across four age groups: younger age (20–39), middle age (40–59), older age (60–89), and extreme old age (100–119 years of age). We first calculated the cell type proportions for each sample across the four age groups (Figure 1A, Supplementary Table S1) and we observed a shift in the distribution of cell proportions from lymphocyte and myeloid cell types from younger ages to centenarians (Figure 1A).

We then calculated the cell type diversity statistic to measure the variability of the proportion of lymphocyte and myeloid cells in each sample (Supplementary Table S2). Comparing the cell type diversity statistics across the four age groups, we found a significant difference in the distribution of the statistics across the four age groups (F-test *p*-value = 0.0001873) (Figure 1B). The increased value of the cell type diversity statistic in the extreme old age group is consistent with the shift in abundances from lymphocytes to myeloid cells, which is an expected change in the immune system with aging (Geiger et al., 2013). We also applied the cell type diversity statistic to measure the variability of the proportions of 12 lymphocyte and myeloid subpopulations in each sample (Supplementary Table S3). We again found a significant difference in the distribution of the statistic in the four age groups (F-test *p*-value = 0.0001875) (Figure 1C). Specifically, centenarians had significantly increased cell type diversity statistics compared to each younger age control group: younger age group (t-test *p*-value = 0.00115), middle age group (t-test *p*-value = 0.00016), and older age group (t-test *p*-value = 0.00363) (Figure 1C). The pattern of the cell type diversity with age groups suggests that centenarians have a more uniform distribution of cell types compared to individuals of younger ages even at a finer resolution of cell types.

The analyses illustrate how the cell type diversity statistic can be used in combination with visualizations of cell type proportions to provide a numerical summary of the distribution of cell types in different conditions. We showed an application of this metric in the context of aging to summarize changes of the distribution of cell types across different age groups, at different resolutions. The metric showed a significant change of the distribution of 12 cell types in extreme old age compared to younger age groups, as well as a significant change of the proportion of lymphocytes and myeloid cells that are biologically relevant to aging (Geiger et al., 2013). Although in our analysis the distribution of the cell type diversity statistics did not change with different cell type resolutions, in other applications the statistic could change since the distribution of the proportions of subpopulations of cells can be very different.

One major challenge in the analysis of single cell transcriptomics data is in the identification and annotation of cell types. There are varying methods to identify cell types (Andrews et al., 2021; Adil et al., 2021; Shekhar and Menon, 2019; Luecken and Theis, 2019) and the resolution of cell type for analysis should be selected based on the biological question of interest (Luecken and Theis, 2019). Another challenge of this type of analyses is accounting for cell types that are not detectable under specific conditions. Other metrics are needed to account for cell types that are not detected in all conditions.

The cell type diversity statistic is applied as a global summary of cell type composition, and additional analyses are required to quantify individual cell type changes and to adjust this analysis for additional covariates. The recent method scCoda uses a Bayesian Dirichlet regression model to examine individuals cell type changes and accounts for the constrained proportions in single cell composition data is particularly promising (Büttner et al., 2021).

Entropy as a metric to study composition level data has been applied in many fields including analyses of microbiome data (Whittaker, 1972; Olde Loohuis et al., 2018; Calle, 2019). The importance in applying this metric to single cell transcriptomics is that it accounts for the constrained proportions of cell types in each sample, and ignoring these constraints can results in inconsistencies when assessing compositional changes (Gloor et al., 2016; Gloor et al., 2017; Calle, 2019; Luecken and Theis, 2019).

## Conclusion

We present the cell type diversity statistic, an entropy-based measure to assess and summarize the overall cell type composition of samples in single cell gene expression data. The diversity statistic allows for the investigation of global cell type compositional changes applicable to studying disease and other conditions at the single cell level. We demonstrate the utility of this method by its application to single cell datasets of aging and extreme old age, and show that it can reveal novel changes in composition in aging at different resolutions.

## Data Availability

Publicly available datasets were analyzed in this study. This data can be found here: The data that support these findings are publicly available and were accessed from several repositories. NATGEN single cell expression data and subject level data were publicly available as referenced in (van der Wijst et al., 2018): https://molgenis58.target.rug.nl/scrna-seq/. PNAS single cell expression data and subject level data was available as referenced in (Hashimoto et al., 2019): http://gerg.gsc.riken.jp/SC2018/. NECS will be available from Synapse (URL https://adknowledgeportal.synapse.org/Explore/Projects/DetailsPage?Grant%20Number=UH2AG064704).

## Code Availability Statement

The cell type diversity statistic is available as a function in R at https://github.com/tanya-karagiannis/Cell-Type-Diversity-Statistic. The function can be applied to a matrix of cell type proportions per sample, a Seurat object, and a Single Cell Experiment object.
