# Supplementary material for: Cell Type Diversity Statistic: An Entropy-Based Metric to Compare Overall Cell Type Composition Across Samples
Source: Front Genet. 2022 Apr 8;13:855076. doi: 10.3389/fgene.2022.855076 (PMC9023789; doi:10.3389/fgene.2022.855076)
Supplement: Supplementary file 1 [file DataSheet1.docx]

**Supplementary Material**

**Cell Type Diversity Statistic: An entropy-based metric to compare overall cell type composition across samples**

**Tanya T Karagiannis^1,2,3^, Stefano Monti^1,3^, Paola Sebastiani^1,2^**

^1^Bioinformatics Program, Boston University, Boston, MA, United States

^2^Institute of Clinical Research and Health Policy Studies, Tufts Medical Center, Boston, MA, United States

^3^Division of Computational Biomedicine, Boston University School of Medicine, Boston, MA, United States

^4^Department of Biostatistics, Boston University School of Public Health, Boston, MA, United States

**Supplementary Methods**

Single cell analysis

Filtering:

After downloading the datasets, we performed all filtering, normalization, and principal component analysis using Seurat v.3 (Stuart *et al.*, 2019). For all datasets, we performed quality control steps based on number of genes and UMIs detected per cell, and percent of mitochondrial genes expressed per cell. For the NECS dataset, we filtered cells with fewer than 200 genes detected and more than 3000 genes detected, and less than 15000 UMI counts detected per cell. We also filtered out cells with more than 15% mitochondrial reads expressed. For the NATGEN dataset, we filtered cells based on similar thresholds from the original manuscript (van der Wijst *et al.*, 2018) with the exception of filtering the number of UMIs per cell greater than 3,500 to remove outlier and doublet cells, and filtering the percent of mitochondrial genes expressed greater than 5 percent to remove damaged or dying cells. For the PNAS dataset, we filtered cells as previously published (Hashimoto *et al.*, 2019).

Normalization, PCA, and Clustering:

After filtering the datasets, we normalized the RNA expression levels of each cell to compare gene expression between sample cells; gene counts for each cell were normalized by total expression, multiplied by a scale factor of 10,000 and transformed to a log2 scale. We then performed PCA analysis based on the top 2,000 highly variable genes detected and clustered cells based on graph-based methods (SNN and Louvain community detection method) (Stuart *et al.*, 2019) based on the top significant PCs for each dataset.

Identification of cell types:

We first identified consensus immune cell types across the two publicly available datasets NATGEN and PNAS. We identified immune subpopulations across each cluster generated from graph based clustering using immune cell type signatures from literature (Chen *et al.*, 2018; Popescu *et al.*, 2019). We calculated the average expression score of each immune cell type signature for each cell to compare the average expression scores of signatures within a cell population. The average expression score of each signature was calculated for a single cell by calculating the average scaled expression of all genes within a signature, with the scaling based on the expression of a control set of genes (AddModuleScore function in Seurat (Stuart *et al.*, 2019), and by taking the absolute value of the average scaled expression to compare scores across signatures within a cell population. In addition, we compared the expression levels of canonical gene markers of these immune populations for comparison (Butler *et al.*, 2018; Hashimoto *et al.*, 2019).

We used a multi-modal approach to identify immune subpopulations in NECS dataset. First, we used the 10 cell-surface protein immune cell marker panel of expression to identify main immune cell types. We then further partitioned the main immune cell types into immune subtypes using graph-based clustering and based on the expression of immune cell type signatures from literature (Chen *et al.*, 2018; Popescu *et al.*, 2019). We calculated the average expression score of each immune cell type signature for each cell as described above to compare scores across signatures within each subtype. In addition, we compared the expression of canonical gene markers of the immune populations identified for comparison (Butler *et al.*, 2018; Hashimoto *et al.*, 2019).

**Supplementary Tables (In SupplementaryData.xlsx)**

**Supplementary Table 1. Proportions of the lymphocyte and myeloid cell types for each sample identified in scRNA-seq data of PBMCs grouped by age group and scRNA-seq data of origin.**

**Supplementary Table 2**. **Cell type diversity statistic for each sample calculated on the proportions of the 2 major lymphocyte and myeloid populations grouped by age group and scRNA-seq data of origin.**

**Supplementary Table 3**. **Cell type diversity statistic for each sample calculated on the proportions of the 12 lymphocyte and myeloid subpopulations grouped by age group and scRNA-seq data of origin.**

**References**

Butler,A. *et al.* (2018) Integrating single-cell transcriptomic data across different conditions, technologies, and species. *Nature Biotechnology*, **36**, 411–420.

Chen,B. *et al.* (2018) Profiling tumor infiltrating immune cells with CIBERSORT. *Methods Mol Biol*, **1711**, 243–259.

Hashimoto,K. *et al.* (2019) Single-cell transcriptomics reveals expansion of cytotoxic CD4 T cells in supercentenarians. *PNAS*, **116**, 24242–24251.

Popescu,D.-M. *et al.* (2019) Decoding human fetal liver haematopoiesis. *Nature*, **574**, 365–371.

Stuart,T. *et al.* (2019) Comprehensive Integration of Single-Cell Data. *Cell*, **177**, 1888-1902.e21.

van der Wijst,M.G.P. *et al.* (2018) Single-cell RNA sequencing identifies celltype-specific cis-eQTLs and co-expression QTLs. *Nature Genetics*, **50**, 493–497.
